# 

**Published:** 1903-07

**Authors:** 


### BOOKS RECEIVED.

A System of Physiologic Therapeutics. A Practical Exposition of the Methods, other than Drug-giving, useful for the Prevention of Disease and in the Treatment of the Sick. Edited by Solomon Solis Cohen, A. M., M. D. Senior Assistant Professor of Clinical Medicine in Jefferson Medical College. Volume X. Pneumotherapy, including Aerotherapy and inhalation Methods and Therapy, by Paul Louis Tissier, Chief of Clinic in the Faculty of Medicine of the University of Paris. Octavo, pp. 494. Illustrated. Philadelphia: P. Blakiston's Son & Co. 1903. (Price for the set, $27.50 net.)

Progressive Medicine. Fifth Annual Series. Volume II., June, 1903. A Quarterly Digest of Advances, Discoveries and Improvements in the Medical and Surgical Sciences. Edited by Hobart Amory Hare, M.D., Professor of Therapeutics and Materia Medica in the Jefferson Medical College of Philadelphia. Octavo, 427 pages, with 46 illustrations. Lea Brothers & Co., Philadelphia and New York. (Per volume, $2.50, by express prepaid. Per annum, in four cloth-bound volumes, $10.00.)

Disease of the Pancreas. Its Cause and Nature. By Eugene L. Opie, M.D., Associate in Pathology in the Johns Hopkins University; Fellow of the Rockefeller Institute of Medical Research. Octavo, pp. 359. Illustrated. Philadelphia and London: J. B. Lippincott Company. 1903. (Price, $3.00.)

A Manual of Refraction and Motility. For Students and Practitioners of Medicine. By William Norwood Suter, M. D., Assistant Surgeon to the Episcopal Eye, Ear and Throat Hospital, Washington, D. C. 12mo, 382 pages, with 101 engravings and 4 colored plates. Lea Brothers & Co., Philadelphia and New York. 1903. (Cloth, $2.00 net.)

Lea's Series of Pocket Textbooks. A Manual of Bacteriology for Students and Physicians. By Fred. C. Zapffe, M. D., Professor of Pathology and Bacteriology in the Illinois Medical College; Professor of Histology in the Department of Medicine and in the School of Dentistry of the University of Illinois, Chicago. 12mo, 350 pages, with 150 engravings and 7 full-page colored plates. Series edited by Bern B. Gallaudet, M. D., Demonstrator of Anatomy and Instructor in Surgery, College of Physicians and Surgeons, Columbia University, New York. Lea Brothers & Co., Philadelphia and New York, 1903. (Price, cloth, $1.50 net; flexible leather, $2.00 net.)

A Textbook of Chemistry for Students of Medicine, Pharmacy and Dentistry. By Edward Curtis Hill, M. S., M. D. Medical Analyst and

Microscopist; Professor of Chemistry and Metallurgy in the Colorado College of Dental Surgery; Professor of Chemistry and Toxicology in Denver and Gross College of Medicine, University of Denver. With 78 illustrations, including 9 full-page half-tone colored plates. Pages XII-523 crown octavo. Philadelphia, F. A. Davis Company, 1903. (Extra cloth, $3.00 net delivered.)

Sexual Impulse in Women. Third Volume in Series. Studies in the Psychology of Sex. By Havelock Ellis, L. S. A. (England), General Editor of the Contemporary Science series since 1899. Sold only to physicians, lawyers, clergymen, advanced teachers, and scientists. Philadelphia. F. A. Davis Company, 1903. (Cloth, $2.00 net, delivered.)

Surgical Asepsis. Especially adapted to operations in the home of the patient. By Henry B. Palmer, M. D., Consulting Surgeon to the Central Maine General Hospital. Ninety illustrations. Page VI-231. Large 12mo. Philadelphia, F. A. Davis Company, 1903. (Price $1.25 net, delivered.)

The Expectant Mother. A treatise on the care of the expectant mother during pregnancy and child-birth and the care of the child from birth to puberty. By W. Lewis Howe, M. D. Pages VIII-63. Small 12mo. F. A. Davis Company, Philadelphia, 1903. ($.50 net, delivered.)

A Textbook of Minor Surgery, including Bandaging. By Newman T. B. Nobles, M. D., Professor of Surgery at the Cleveland Homeopathic Medical College. 325 pages. Boericke & Tafel, Philadelphia, 1903. (Cloth, $2.50; Postage, 15 cents.)

The Practical Application of the Roentgen Rays in Therapeutics and Diagnosis. By William Allen Pusey, A. M., M. D., Professor of Dermatology in the University of Illinois; and Eugene W. Caldwell, B. S., Director of the Edward N. Gibbs *x*-Ray Memorial Laboratory of the University and Bellevue Hospital Medical College, New York. Handsome octavo volume of 591 pages, with 180 illustrations, nearly all clinical. W. B. Saunders & Co., 1903. (Cloth, $4.50 net; Sheep or Half Morocco, $5.50 net.)

A Textbook of Modern Materia Medica and Therapeutics. By A. A. Stevens, A. M., M. D., Lecturer on Physical Diagnosis in the University of Pennsylvania; Physician to the Episcopal and St. Agnes Hospitals, Philadelphia. Third edition, greatly enlarged, rewritten, and reset. Handsome octavo of 663 pages. W. B. Saunders & Company, 1903. (Cloth, $3.50 net.)

Transactions of the American Roentgen Ray Society. Third annual meeting held at Chicago, Ill., December 10 and 11, 1903. James B. Bullitt, M. D., Secretary. (Price, cloth $1.25; paper, 75 cents.)

Twenty-Second Annual Report of the State Department of Health of New York, for the year ending December 31, 1901. With maps. J. B. Lyon Company, Albany, State printers, 1902.

A Manual of Surgical Treatment. By W. Watson Cheyne, M. B., F. R. C. S., F. R. S., Professor of Surgery in King's College, London; Surgeon to King's College Hospital, etc.; and F. F. Burghard, M. D. and M. S. (Lond.), F. R. C. S., Teacher of Practical Surgery in King's College, London; Surgeon to King's College Hospital, etc. In seven imperial 8vo volumes, with illustrations. Volume VII, 582 pages with 113 illustrations. The treatment of the surgical affections of the rectum, the liver, pancreas and spleen, the genitourinary organs, the breast and the thorax. Philadelphia and New York. Lea Brothers & Co. 1903. (Price, $5.75.)

Twentieth Century Practice. An International Encyclopedia of Modern Medical Science by Leading Authorities of Europe and America. Edited by Thomas L. Stedman, M. D., New York City. In 21 volumes.

Volume XXI.—Supplement. New York: William Wood & Co. 1903. (Price, cloth, $5.00.)

Surgical Diseases of the Abdomen. With special reference to diagnosis. By Richard Douglas, M. D. Formerly Professor of Gynecology and Abdominal Surgery, Medical Department, Vanderbilt University, Nashville. Octavo, pp. 883. Illustrated by 20 full-page plates. Philadelphia: P. Blakiston's Son & Co. 1903. (Price, cloth, gilt top, $7.00.)

### LITERARY NOTES.

### Index to New Series Volume XLII.

### (Whole Number Volume LVIII.)

AUGUST, 1902 – JULY, 1903.

PAGE.

ACETANILID POISONING ..129
Acetezone in Gastrointestinal Diseases ................676
Acetezone in Typhoid Fever, 40, 284, 915
Adrenalin, Experiments with .......128
Anemia, Pepto-Mangan in..........434
Anesthesin ........................ 44
Antitoxin from the State Laboratory.448
Argyrol, Methods of Using ........243
Arthritis, Salt Pack in Rheumatoid..514
Asthmatic, Diet of the ............317
Auerbach, M. A., Gout and its Treatment..................817
Summer Diarrheas of Children.............. 37

BARNES, A. C., Methods of Using Argyrol ....................243
Bayliss, A. W., Thoughts on Modern Medicine ..........891
Beahan, A.L., The Surgical Specialty.313
Benedict, A. L., Water Supply and Sewerage of Buffalo ............869
Benninghoff, G. E., Surgical Tuberculosis ........................256
Bile as a Solvent..................120
Variations in Composition of...121
Bissell, William G., Does the Public Telephone Transmit Disease? ....408
Bladder Drainage .................910
Wall, Non-Permeability of..123
Blood, Changes in Chemical Composition of ..................121
Examinations in General Practice .....................808
in Relation to Surgical Diagnosis ....................174
Books Received ...75, 150, 229, 311, 385, 466, 542, 625, 705, 784, 866, 946
Breech Presentations, Management of 417
Bronchitis, Acute .............132, 410
Infantile ...............132
with Bronchoplegia......428
Buffalo Review Exposing Antonius..448
Burke, Joseph, Congenital Pulmonary Stenosis ....................15, 92

PAGE.

Buttermilk, a Food for Infants......911
Button, C. A., Menstruation........182

CANCEROUS EPITHELIUM, Evidence of Nuclear Division of Cell Inclusions in..........736
Cardiac or Renal Dropsy...........912
Caput Coli, Surgical Importance of..332
Carbolic Acid Poisoning ...........127
Treatment of. 43
Catarrh, Gastrointestinal, Cold Compresses in ......................397
Children, Injuries and Infections of Newly-born............904
Law to Regulate Employment of...............926
Management of Rheumatic 203
Clark, Joseph C., Electricity in Medicine and Surgery................511
Climate of the West, Observations on. 336
Clitoris, Cancer of................108
Cocaine, How to Keep in Solution..194
Collargolum in Infectious Diseases..681

**College and Hospital Notes:**

Buffalo Children's Hospital ....457
Buffalo Clinical Laboratory.....220
Buffalo General Hospital...859, 936
Buffalo Hospital Sisters of Charity ........................530
Buffalo Marine Hospital .......696
Cincinnati College of Medicine and Surgery................219
Denver and Gross College of Medicine .................. 66
Emergency Hospital.......220, 530
Infirmary, Dr. McMurtry's......858
Jackson Memorial Hospital ....373
Lexington Heights Hospital....530
Michigan University Hospital, Ann Arbor.................373
New York School of Clinical Medicine...................532
New York Skin and Cancer Hospital.......................457
Olean General Hospital........531

PAGE.
Resident Physicians at Buffalo Hospitals....859
Riverside Hospital....456
Rockefeller Institute....618
Smallpox Hospital at Batavia...457
Tuberculosis Hospital at New Haven....457
University of Buffalo, Medical Department.66, 220, 297, 530, 775
Washington Post-Graduate Medical School....531
Colton, A. J., Infantile Eclampsia...731
Infant Feeding....324
Congdon, Charles E., Cancer of the Clitoris....108
Immediate Repair of Soft Parts following Labor .401
Constipation, Electricity in Habitual.356
Convulsions of Obscure Origin....912

**Correspondence:**

Dangers of Alcohol—T. D. Crothers....683
Maltine Company Prize Essay Awards— C. C. Heuman....358
Physicians' Club—B. H. Grove.599
Prophylaxis of Venereal Diseases —Ludwig Weiss....682
William Beaumont, Memorial Address on — Robert Luedeking....133
Cough, Clinical Memoranda on....881
Coughs and Their Treatment....522
Creosote and Creosotal in Pneumonia. 521
Creveling, J. P., Surgical Importance of the Caput Coli, etc....332
Cumston, Charles Greene, Henry de Mondeville, the Man and His Writings....486, 549, 642
Cystitis, Treatment of Chronic....764

DAGGETT, B. H., Study of the Urine without a Microscope.195
Davie, Carey D., Medical Expert Testimony....566
Deafness, Points in the Treatment of Catarrhal....389
De Mondeville, Henry, the Man and His Writings....486, 549, 642
Delegates, House of, A. M. A..843, 844
Diarrheas of Children, Summer.... 37
Diehl, Alfred E., A Case of Cutaneous Horn....345
Doane, Bishop, Election of as Chancellor....449
Dorr, L. Bradley, Antistreptococcus Serum in Erysipelas.... 44
Dowd, J. Henry, To what Extent may Female Genitals be Saved from Mutilation?....398

PAGE.
Dunham Home....214
S. A., Cerebral Hemorrhage.192
Secondary Traumatic Insanity....233
Dunning, L. H., Carcinoma of the Cervix Uteri....411
Dysmenorrhea, Clinical Note on....133
Treatment of....359

ECLAMPSIA, Infantile....731
Electricity in Medicine and Surgery....511
Epistaxis, Suprarenal Extract in....200
Ergoapiol in Diseases of the Female. 50
Erysipelas Treated with Antistreptococcus Serum.... 44
Treatment of....127
Everybody's Magazine....851
Examinations, State Medical Licensing....692

FARBENFABRIKEN of Elberfeld Company and Substitution....852
Feeding, Infant....324
Felsin in Dyspepsia....919
Filtering Plant....525
Forsyth, E. A., Intratracheal Medication....187
Paraffin in Correction of Nasal Deformities....836
Fowler, George Ryerson, Provisional Hemostasis in Operations on Head and Neck....789
Fronczak, F. E., Treatment of Carbolic Acid Poisoning.... 43

GASTRIC CATARRH, Treatment of Chronic....130
Gastric Juice, Digestive Power of....122
Gaylord, Harvey R., Evidence of Nuclear Division of Certain Cell Inclusions in Cancerous Epithelium..736
Genitals, May Female be Saved from Mutilation?....398
Goldberg, Sigmund, Why Minor Gynecological Operations Fail in Results....875
Gout and its Treatment....817
Grand Jury and the Newspapers....690
Gynecological Operations, why Minor Fail in Results....875

HAAS, LEOPOLD F. W., Clinical Memoranda on Cough..881
Hall, A. L., Hay Fever, Treatment of.... 52
Radical Operation for Femoral Hernia....407

PAGE.

Headaches, Migrainin in ...........438
Heart, Surgery of..................282
Heffron, John L., Observations on the Climate of the West.........336
Hematuria ..........................125
Hemorrhage, Cerebral .............192
Hemostasis, Provisional in Operations on Head and Neck..............789
Hernia, Radical Operation for Femoral ............................407
Herpes Zoster, Hypodermics for ....913
Hippuric Acid Metabolism in Man ..122
Hopkins, Henry Reed, Progress, Liberty, Unity—Presidential Address .469
Horn, A Case of Cutaneous........345

ICE-BAG, Caution in Use of......840
Infant, Mutilation of Newborn..213
Infants, Acute Articular Rheumatism in .............204
Feeding of, Premature..201
Summer Diarrhea of ...203
Infections, Therapy of Puerperal and Surgical ........................838
Insane, Diagnosis of General Paralysis of...........................723
Insanity, Secondary Traumatic......233
Toxins of...............629
Insomnia of Pulmonary Tuberculosis, Treatment of....................179
Intestine, Removal of 265 cm. of Gangrenous ......................637
Intratracheal Medication...........187
Iron as a Remedy..................593
Ithaca, Typhoid Fever at ..........607

**Illustrations:**

Congdon: Cancer of the Clitoris.109
Davis, William E. B., portrait ..613
Diehl: Cutaneous Horn........346
Dunham: Secondary Traumatic Insanity.
Fig. 1, Photograph of Patient.................234
Fig. 2, Diagram of a Neuron ..............235
Fig. 3, Cells of Cerebral Cortex...............236
Fig. 4, Section of Cerebellar Cortex............237
Forsyth: Paraffin in Nasal Deformity.
Fig. 1, Before Injection..837
Fig. 2, After Injection...837
Gaylord: Cancerous Epithelium.
Figs. 1 to 21 .......738–755
Plate with 8 figs., inset, facing ...............760
Krauss: Medico-Legal Cases.
City Elevator B.........831

PAGE.

Long: Stimulation.
Plate, 8 figs., showing pulse tracings.........806
Lorenz, Adolf, portrait.........443
Lothrop, Thomas, portrait .....135
Love, I. N., portrait ...........931
Myers: Calcareous Tumor Degeneration ...........510
Mynter, Herman, portrait ......611
Potter: Century of Medical History.
Joseph C. Green, portrait..261
W. E. Robbins, " ..263
Elias T. Dorland, " ..265
Lewis P. Dayton, " ..266
Frank W. Abbott, " ..267
Samuel G. Dorr, " ..269
Wyeth & Brother: Lynch Aseptic Syringe Container..598

**Instruments, New:**

Syringe Container, Lynch Aseptic—Wyeth & Brother .......598

**Items:**

Annals of Surgery ..............468
Antikamnia Calendar, 1903.....468
Antikamnia Chemical Co., Removal ......................787
Antiphlogistine Christmas Folder ..........................468
Daily Reminder Calendar, 1903—Scott & Bowne ...........468
Documents in the Case ........548
Enno Sander Prize............548
Fairchild Brothers & Foster Obtain Conviction for Substitution .......................788
Illinois Central Railroad .......707
Lambert Pharmacal Co........548
Mariani's Album of Physicians..708
Matthews, Lewis S. & Company.628
Medical Corps, U. S. Navy.....788
Mellier Drug Company Art Plaque.....................868
New York Pharmacal Association Calendar, 1903..........468
N. Y. Pharmacal Association...708
Oppenheimer Institute.....708, 787
Physicians' Daily Memorandum, 1903.......................468
Poisons and Antidotes—Maltine Company ..................948
Polk's Medical Register........548
Queen and Crescent Route to New Orleans ...............787
Rudolf Virchow, Monument to .388
Tarrant & Company...........152

JACK, GEORGE N., Diet of the Asthmatic ....................317

PAGE.

KENDALL, W. P., Experiences in Philippine Islands....... 83
Kidney, Cysts of ............436
Kidney, Stone in..................204
Koumyss, Preparation of...........425
Krauss, William C., Report of Two Medico-Legal Cases .............829

LABOR, Management of Premature .....................903
Lacerations, Immediate Repair of After Labor............401
Lead Poisoning, Unusual Causes of.393

**Leading Articles:**

American Medical Association at New Orleans ...............688
Antonius, Exit................765
Boston Medical and Surgical Journal, 75th Anniversary....689
Carcinoma in Women .........139
Charaka Club, Proceedings of ..523
Charter of the Medical Society of the State of New York ...439
Code of Ethics...............602
Conference Committees on Medical Unity...................360
Death, Manual of International Classification of Causes of ...925
Director of Charities and Correction in Philadelphia.......848
Drug Substitution Again...... 605
Expert Testimony.............603
Grocery Druggists.............362
Hospitals for the Psychopathic .288
Is it Words or Things? ........141
Journals, Consolidation of two Prominent .................921
Long Live King Edward VII... 53
Lorenz, Dr. Adolf.............442
Lothrop, Thomas .............134
Medical Bureau, Pan-American Exposition .................767
Medical Society of the State of New York ................600
Medical Unity ................137
Medical Ethics, Principles of...922
Merritt Bill, Regents, etc.......606
Newspapers, Two Buffalo...... 55
Osteopathy, Doctor of.........212
Panama Canal: How Wood Would Build It.............211
Passing of a Fad .............363
Pension Bureau and the G.A.R..286
Progress Toward Medical Unity.841
Reciprocity in Licensure Once More .....................445
Reinstatement of Drs. Heath and May ....................... 56
Rubber-Stamp-Circular Degrees.844

PAGE.

State Educational Systems.....686
University of Buffalo..........847
Virchow .....................207

**Literary Notes:**

American Journal of Dermatology and Genitourinary Diseases .....................785
Archives of Pediatrics.........545
Batavia Woman's Hospital.....948
Battle & Co., Colles's Fracture. Chart No. 12...............948
Buffalo State Hospital, Annual Report.....................387
Bulletin of the Free Hospital for Women ................707
Bulletin, The .................545
California State Journal of Medicine ......................544
Dallas Medical Journal ........544
Dental Materia Medica, Therapeutics and Prescription Writing .......................706
Diabetes Mellitus — Charles Roome Parmele Co...........387
Diseases of the Nervous System and Muscles — Antikamnia Chemical Co...............867
Gould's Biographic Clinics (Blakiston).....................467
Hot Springs Medical Journal...311
Index Medicus ...............545
Internal Secretions and Principles of Medicine............626
International Medical Magazine.868
J. B. Lippincott Company, New Building ...................151
Journal of Cutaneous Diseases and Syphilis...............544
Journal of Michigan State Medical Society .................311
Journal of Tuberculosls .......627
La Revue Medicale du Canada .544
Lea Brothers & Co., Announcement ......................868
Lloyd's Famous Satires........628
Mariani's Coca Leaf...........388
Medical Book News....... 76, 546
Medical Critic Index Medicus ..868
Medical Library Journal........545
Medical Standard .............151
Medicine.....................545
Medico-Legal Bulletin .........312
Modern Medical Preparations...627
Neoferrum—Maltine Company .387
New York Medical Journal.....467
Northwest Medicine...........627
Old Dominion Journal of Medicine and Surgery............ 76
Providence Retreat Report.....785

PAGE.

Quarantine Sketches — Maltine Company .................. 867
Report of Niagara Falls Department of Health .............. 627
Report of the Charity Eye, Ear and Throat Hospital ........ 707
Samuel D. Gross Prize ......... 867
Standard Medical Directory, 1903 ...................... 706
State Hospital for Crippled and Deformed Children .......... 628
Trained Nurse and Hospital Review ...................... 544
Tribune Review ................ 543
W. B. Saunders & Co. .......... 546
W. B. Saunders & Co., New Books ..................... 786
Wichita Medical Journal ....... 388

Liver, Hydatid Cyst of ............ 121
Percussion of ................ 285
Multiple Abscess of .......... 901
Long, Eli H., Stimulation .......... 799
Lorenz, Adolf, Departure of ........ 525
Lothrop, Thomas, In Memoriam .... 117
Lyon, Irving Phillips, Blood Examinations in General Practice ......... 808

MACPHERSON, J. D., Popular Medical Education in Limiting Spread of Disease ...... 709
Malaria, Chronic .................. 131
Maltine Company's Prizes .......... 365
Mansperger, William H., Tuberculosis of the Peritoneum .......... 249
Mason, William H., In Memoriam ... 908
McGuire, Francis W., Causes of Suppuration ...................... 583
McKinley, President, Assassin's Mental Condition .................. 608
Medical Editors' Dinner ........... 844
Education in Limiting Spread of Disease ............. 709
History of the County of Erie .................. 260
Medicine, Thoughts on Modern ..... 891
Medico-Legal Cases, Report of ..... 829
Menstruation, Normal and Abnormal . 182
Mental Diseases, Detention Hospital for Acute ...................... 33
Metabolism, Treatment of Diseases of ........................... 917

**Miscellany:**

Arlington Chemical Co. ......... 312
Battle & Company's Surgical Charts. No. 9, 232; No. 10, 548; No. 11, 707; No. 12 .... 948
Children's Hospital, Report .... 547
Civil Service Examination ...... 547
Dr. Bissell's Laboratory Course . 312
Dr. Pitkin's Finsen Apparatus .. 312
Maltine Company Essays ....... 231
Mattison Prize ............... 232
Medical Corps of the Army .... 547

Myers, J. F., Uterine Fibroids ...... 504
Mynter, Herman, In Memoriam ..... 677

NASAL DEFORMITY, Paraffin in Correction of ........... 836
Nephropexy by a New Method . 433
Nephroureterectomy, Pregnancy and Labor Following ................ 596
Neuralgia, Alkalies in ............... 599
New Orleans, Attendance at ........ 844
Newspaper Medicine .............. 57
Nurses, Graduation of at Havana ... 607
Literature Class for ........ 690
State Examination of ...... 927

**Obituary:**

Asch, Morris J ................ 293
Bancroft, Frederick J .......... 528
Benjamin, M. N ............... 452
Boysen, Theodore H ........... 855
Bryson, John P. .............. 927
Byrne, John .................. 293
Daniels, Clayton M ............ 215
Davis, William E. B. .......... 612
Deane, William J .............. 528
Foster, Hubbard A. ........... 933
Fritz, William C. ............. 772
Gantt, Ephraim W. ............ 528
Gay, Sarah A. ................ 695
Grissom, Eugene .............. 60
Hixon, Levi J ................. 451
Homans, John ................ 615
Hooper, P. O. ................ 143
Howard Chester .............. 933
James, Bushrod W ............ 528
James, Harvey L ............. 773
Jelks, James Thomas .......... 59
Jenks, Edward W. ............. 773
Kitchel, L. H. ................ 933
Kraus, Jacob M ............... 450
Krombien, Louis E ............ 452
Laine, Joseph R .............. 528
Lewis, Tousley B .............. 616
Little, Edward ................ 368
Love, Isaac Newton. .......... 930
Mason, William H. ............ 908
McBeth, John ................ 855
McCann, Thomas ............. 934
Miller, Guy Bryan ............ 856
Miller, John ................. 694
Miner, Mary C. ............... 695
Miner, Worthington C. ......... 856
Morton, Thomas G. ........... 857
Mynter, Herman .............. 611
Newell, Jared M ............... 933
Oliver, William ............... 60

PAGE.

Osborne, Charles A. . . . . . . . . . . . 528
Phelps, Abel Mix . . . . . . . . . . . . . 292
Potter, Alice M. . . . . . . . . . . . . . 933
Putnam, James O. . . . . . . . . . . . . 788
Reed, David H. . . . . . . . . . . . . . . 616
Reed, Walter . . . . . . . . . . . . . . . . 367
Sänger, Max . . . . . . . . . . . . . . . . 616
Slocum, George D . . . . . . . . . . . . . 616
Storck, Eugene E. . . . . . . . . . . . . 216
Thomas, T. Gaillard . . . . . . . . . . . 694
Townsend, Martin I. . . . . . . . . . . . 695
Wishart, J. Wilson . . . . . . . . . . . . 60

ODELL, GOVERNOR, and State Medical Society . . . . . . . . . . . . . 365
Ophthalmia Neonatorum, Credé's Treatment of . . . . . . . . . . . . . . . . . 685
Osteopaths in Alabama . . . . . . . . . . . . 56

PARK, ROSWELL, Removal of 265 cm. of Gangrenous Intestine . . . . . . . . . . . . . . . . . . . . . . 637
Pension Examining Surgeons' Reports, Address on . . . . . . . . . . . . . . 153
Peptic Activity, Method of Estimating . . . . . . . . . . . . . . . . . . . . . . . . 120
Peritoneum, Tuberculosis of . . . . . . . . 249

**Personal:**

Brown, Ira C.; Glosser, H. H.; Lorenz, Adolf . . . . . . . . . . . . . . . 450
Carpenter, Thomas B.; Mikulicz, Professor; Chester, Charles O.; Grant, Sir James; Francis, Charles S. . . . . . . . . . . . . . . . . . . 772
Carroll, Jane W.; Jelks, F. W.; Mann, Matthew D. . . . . . . . . . . . 142
Clayton, Mary; Marsh, Marion; Lorenz, Adolf; Mynter, Herman . . . . . . . . . . . . . . . . . . . . . . 292
Clinton, Marshall; Smith, Eugene; Wilcox, Dewitt G.; Danser, E. G.; Moseley, George T.; Crego, Floyd S.; McKelway, St. Clair . . . . . . . . . . . . . . . . . . . 609
Constantine, D. J.; Urban, Adolph H.; Gregory, Willis G.; Mead, Eva V.; Hagen, Beatrice Todd; Granger, R. B. 527
Douglas, Richard; Griffith, Jefferson D.; Dunning, L. H.; Walker, Edwin; Musser, John Herr; Benedict, A. L.; Charters, James W.; Hussey, E. P.; Keiser, Max; Sohmer, A. E. . . 929
Fowler, George Ryerson; Woodbury, John McGaw; Greene, Walter D.; Clark, Edward; Ballou, E. H.; Satterlee, Richard H. . . . . . . . . . . . . . . . . . . . . 693

PAGE.

Gaylord, Harvey R.; Grant, John H. . . . . . . . . . . . . . . . . . . . . . . . 143
Griswold, V. M.; Peterson, Frederick; Forwood, William H.; Dunham, Sydney A. . . . . . . . . . . 214
Grosvenor, Joseph W.; Lewis, F. Park; Davis, T. D.; McIntire, Charles; Garcelon, Alonzo . 930
Hall, Winfield S; Frost, E. L; Le Breton, Prescott; Doty, Alvah H. . . . . . . . . . . . . . . . . . . 610
Harrington, D. W.; Putnam, James O.; Pohlman, Angustus; Blaauw, E. E.; Freeman, S. A.; Cary, Charles; Elsner, Henry L.; Rogers, B. F . . . . . . . . . . . . 770
Himmelsbach, G. A.; Gaylord, Harvey R.; Crockett, Montgomery A.; Clowes, G. H. A, Smith, Chauncey Pelton; Preiss, Frederick . . . . . . . . . . . . 928
Macdonald, W. G.; Forsyth, Edgar A; Wilson, Nelson W; Meisburger, William; Crockett, Montgomery A.; Hayd, Herman E. . . . . . . . . . . . . . . . . . 771
McMurtry, L. S.; Gaylord, H. R.; Smith, Chauncey Pelton; Wasdin, Eugene . . . . . . . . . . . . . . . . 855
Millener, F. H.; Krauss, Wm. C. 526
Moore, William A.; Pohlman, Julius; Lewi, M. J.; Park, Roswell; Parsons, James Russell, Jr.; Lewis, Daniel; Grant, John H . . . . . . . . . . . . . . . . . . . . 59
Mulford, Henry J.; Chalmers, John; Breuer, Max C.; Barrows, Franklin W. . . . . . . . . . . . 853
O'Neill, J. M.; Lewi, M. J; Bayliss, A. W.; Rowe, Alice E.; Orth, Johannes; Mansperger, W. H. . . . . . . . . . . . . . . . . . . . . 215
Phillips, Wendell C.; Daniels, John H.; Calkins, G. N.; Mickle, Herbert; Johnson, Burt C.; Mead, Harry . . . . . . . 692
Preiss, Frederick . . . . . . . . . . . . . . 608
Putnam, James O.; Mann, M. D.; Lewis, Daniel; Reed, Charles A. L.; Rhodes, John E.; Keyes, Regina Flood . . . . . . . . . . . . . . . 291
Satterlee, Richard H.; Ryerson, G. Sterling; Lydston, G. Frank; Carpenter, Frank L.; Andrews, Charles H. . . . . . . . . . . . . . . . . . 449
Simmons, George H.; Forwood, William H.; O'Reilly, Robert M; Rolph, R. T.; Croom, Sir John Halliday . . . . . . . . . . . . . . . 58
Smith, Chauncey Pelton; McAdam, L. J . . . . . . . . . . . . . . . . . 694

PAGE.

Trowbridge, Grosvenor R.; Burke, Joseph; Gnichtel, A. L.; Cott, George F.; Satterlee, R. H.; Gibson, James A.; Ewald, Prof. C. A.; Stockton, Charles G. . . 854
Waterman, J. Hilton; Ring, Charles A.; Bissell, Wilson S.; Stucky, Thomas Hunt; Walker, Edwin; LeBreton, Prescott . 367
Wolcott, Edwin Henry; House, William; Clausius, M. F; Gaylord, Harvey R . . . . . . . . . . . . 366

Peterson, Frederick, In the Shade of Ygdrasil . . . . . . . . . . . . . . . . . . . . . . 769
Phelps, William C., Experience with Gunshot Wounds in Civil Life . . . . 11
Philippine Islands, Experiences in . . . 83
Physicians' League, work of . . . . . . . . 29
Plague Invasion of Pacific Coast . . . . 608
Pneumonia, Creosote in . . . . . . . . . . . . 596
Leukocytosis in . . . . . . . . 597
Potter, William Warren, Century of Medical History in the County of Erie . . . . . . . . . . . . . . . . . . . . . . . . . . 260
Practitioners, Unqualified and Criminal Negligence . . . . . . . . . . . . . . . . . . 357

**Progress in Medical Science:**

Genitourinary and Syphilitic Diseases, by Byron H. Daggett . . . . . . . . . . 123, 204, 430, 910
Pediatrics, by Maud J. Frye . . . . 201
Physiological Chemistry, by John A. Miller . . . . . . . . . . . . . . . . . . . 120
Therapeutic Notes, by Nelson W. Wilson . . . . . . . . . 127, 425, 911

Progress, Liberty, Unity . . . . . . . . . . . . 469
Pulmonary Apex, Method of Examining . . . . . . . . . . . . . . . . 920
Stenosis, Congenital . . . 15, 92
Tuberculosis, Ichthyol in . 129
Purpura Hemorrhagica, Therapy of . . 837
Putnam, James Wright, Toxins of Insanity . . . . . . . . . . . . . . . . . . . . . . . . . 629

RAUB, J. F., Address on Reports of Pension Examining Surgeons . . . . . . . . . . . . . . . . . . . . . . 153
Reed, Charles A. L., And the Code of Ethics . . . . . . . . . . . . . . . . . 843
Rudolf Virchow, an Appreciation . . . . . . . . . . . . . . . . . . . . . 656
Regents, Appointments by . . . . . . . . . 853
Reisman, S. C., Treatment of Insomnia of Pulmonary Tuberculosis . . . . 179
Resolutions by New York State Association . . . . . . . . . . . . . . . . . . . . 447

PAGE.

**Reviews:**

Abbott: Manual of Bacteriology . 226
Albert: Diagnosis of Surgical Diseases . . . . . . . . . . . . . . . . . . . 73
Allen: Practitioner's Manual . . . 307
Bacon: Manual of Otology . . . . . 310
Blakiston: Visiting List for 1903 . 383
Brubaker: Compend of Physiology . . . . . . . . . . . . . . . . . . . . . 623
Buck: Reference Handbook of Medical Sciences, Vol. V . . . . 536
Butler: Materia Medica, Therapeutics, etc . . . . . . . . . . . . . . . . . 777
Byford: Manual of Gynecology . 538
Cathell: The Physician Himself . 700
Chapin: Infant Feeding . . . . . . . 221
Chase: General Paresis . . . . . . . . 381
Cheyne-Burghard: Manual of Surgical Treatment, Vol. VI . . 225
Cohen: System of Physiologic Therapeutics, Vol. IX., 70; Vol. V . . . . . . . . . . . . . . . . . . . . . 534
Crile: Problems in Surgical Operations . . . . . . . . . . . . . . . . . 145
Crocker: Diseases of the Skin . . 859
Culbreth: Materia Medica and Pharmacology . . . . . . . . . . . . . . 779
Cushny: Pharmacology and Therapeutics . . . . . . . . . . . . . . . 865
Davis: Mother and Child . . . . . . 228
Davis: Principles and Practice of Bandaging . . . . . . . . . . . . . . . 374
Deaver: Surgical Anatomy . . . . . 942
Doty: Prompt Aid to the Injured . . . . . . . . . . . . . . . . . . . . . 309
Dudley: Principles and Practice of Gynecology . . . . . . . . . . . . . . 459
Eckley: Practical Anatomy . . . . 701
Einhorn: Diseases of the Stomach . . . . . . . . . . . . . . . . . . . . . . 943
Eyre: Bacteriological Technique . 537
Fox: Practical Treatise on Smallpox . . . . . . . . . . . . . . . . . . . . . . 72
Gant: Diseases of Rectum and Anus . . . . . . . . . . . . . . . . . . . . . 460
Garrigues: Textbook of Obstetrics . . . . . . . . . . . . . . . . . . . . . . . 619
Gerrish: Textbook of Anatomy . 461
Gibson-Russell; Physical Diagnosis . . . . . . . . . . . . . . . . . . . . . 375
Gleason: Diseases of the Ear . . 540
Gorham: Laboratory Course in Bacteriology . . . . . . . . . . . . . . . . 147
Gould: Biographic Clinics . . . . . 699
Gould: Year Book of Medicine and Surgery, 1903 . . . . . . . . . . . 862
Gradle: Diseases of Nose, Pharynx and Ear . . . . . . . . . . . . 68
Graham: Massage . . . . . . . . . . . . 779
Grant: Surgical Diseases of Face, Mouth, etc . . . . . . . . . . . . 458

PAGE.
Grayson : Throat, Nose and Ear. 302
Grindon : Diseases of the Skin . . 862
Grunwald - Newcomb: Mouth Pharynx and Nose . . . . . . . . . . . 941
Guenther : Epitome of Physiology . . . . . . . . . . . . . . . . . . . . . . 781
Hadra : Public and the Doctor . . 624
Hare : Practical Diagnosis . . . . . 459
Hare; Progressive Medicine, Vol. II., 1902, 75; Vol. III., 305; Vol. IV., 539; Vol. I., 1903 . . . . . . . . . . . . . . . . . . . . . . 865
Hare: Textbook of Practical Therapeutics . . . . . . . . . . . . . . . 306
Harrington: Manual of Hygiene. 939
Hatfield : Diseases of Children . . 863
Head: Practical Medicine Series, First Series—
Vol. IV., Gynecology . . . . . . . . 72
Vol. V., Obstetrics . . . . . . . . . . 226
Vol. VI., General Medicine . . . 227
Vol. VII., Materia Medica, etc. 305
Vol. VIII., Pediatrics and Orthopedic Surgery . . . . . . . . . . 541
Vol. IX., Physiology, Pathology, etc . . . . . . . . . . . . . . . . 379
Vol. X., Skin, Venereal Diseases, Nervous and Mental Diseases . . . . . . . . . . . . . . . . . 783
Second Series—
Vol. I., General Medicine . . . . 622
Vol. II., General Surgery . . . . . 699
Vol. III., Eye, Ear, Nose and Throat . . . . . . . . . . . . . . . . . . . 782
Vol. IV., Gynecology . . . . . . . . 945
Helferich : Atlas of Fractures and Dislocations . . . . . . . . . . . . 377
Hemmeter : Diseases of the Stomach . . . . . . . . . . . . . . . . . . . 375
Henry : Practical Gynecology . . . 374
Herter : Chemical Pathology in re. Practical Medicine . . . . . . . . 71
Holt : Diseases of Infancy and Childhood . . . . . . . . . . . . . . . . . 309
Hopkins : The Roller Bandage . . 307
Howard : The Perverts . . . . . . . . 222
Huntington ; Abdominal Anatomy . . . . . . . . . . . . . . . . . . 776
Jacobson : Operations of Surgery, 2 vols . . . . . . . . . . . . . . . . 223
Janet : Mental State of Hystericals . . . . . . . . . . . . . . . . . . . . . . . 69
Jewett : Childbed Nursing . . . . . . 74
Johnson : First Aid Manual . . . . 150
Keay : Medical Treatment of Gallstones . . . . . . . . . . . . . . . . . . 228
Kelsey : Surgery of the Rectum . 149
Keyes: Surgical Diseases of Genitourinary Organs . . . . . . . . . . . . 944
Koplik : Diseases of Infancy and Childhood . . . . . . . . . . . . . . . . . . 378

PAGE.
Lea : Medical News Visiting List for 1903 . . . . . . . . . . . . . . . . . . . 384
Le Fevre : Physical Diagnosis . . 465
Leroy : Essentials of Histology. 864
Leuf : Practical Gynecology . . . . 697
Lippincott: International Clinics, Twelfth Series—Vol. I., 74; Vol. II., 308; Vol. III., 464 Vol. IV . . . . . . . . . . . . . . . . . . . 701
Manton : Manual of Obstetrics . . 783
Mathews : How to Succeed in Practice . . . . . . . . . . . . . . . . . . . 532
Mattison : Treatment of Morphinism . . . . . . . . . . . . . . . . . . . 702
McGrath: Anatomy and Surgery. 778
McMurrich : Developmeut of Body . . . . . . . . . . . . . . . . . . . . . 700
Medical Directory of New York, New Jersey and Connecticut . . 540
Morton : Genitourinary Diseases and Syphilis . . . . . . . . . . . . . . . . 67
Murray : Quain's Dictionary of Medicine . . . . . . . . . . . . . . . . . . . 148
Norris : American Textbook of Obstetrics . . . . . . . . . . . . . . . . . 623
Nothnagel : Encyclopedia of Medicine, Vol. III., 303; Vol. IV . . . . . . . . . . . . . . . . . . . . . . 622
Ochsner : Clinical Surgery . . . . . 620
Ochsner : Handbook of Appendicitis . . . . . . . . . . . . . . . . . . . . . . 71
Ortel : Medical Microscopy . . . . . 624
Ostrom : Massage and Swedish Movements . . . . . . . . . . . . . . . 306
Phillips : Spectacles and Eyeglasses . . . . . . . . . . . . . . . . . . . . 465
Posey-Wright: Eye, Nose, Throat and Ear . . . . . . . . . . . . . . . . . . . 533
Potter, Materia Medica, etc . . . . 539
Reese : Medical Jurisprudence . . 624

Reports :

Commissioner of Education, Vol. I, 1900–01 . . . . . . . . . 702
Johns Hopkins Hospital, Vol. X . . . . . . . . . . . . . . 465, 782
New York State Board of Health, 1900 . . . . . . . . . . . 228
Pennsylvania State Board of Health, Vols. I. and II., 1900 . . . . . . . . . . . . . . . . . . . 150
Presbyterian Hospital, New York, Vol. V . . . . . . . . . . . 382

Reynolds-Newell : Practical Midwifery . . . . . . . . . . . . . . . . . . . . 535
Rockwell: Textbook of Anatomy. 703
Rockwell-Dana : Kirke's Physiology . . . . . . . . . . . . . . . . . . . . . 540
Rose-Carless : Manual of Surgery . . . . . . . . . . . . . . . . . . . . . . . 537

PAGE.

Rossiter: Story of a Living Temple .....................705
Sajous: Internal Secretions and Principles of Medicine.......937
Savage: Ophthalmic Myology ..310
Schalek: Diseases of the Skin..463
Schleif: Materia Medica, Therapeutics, etc..................541
Schmaus-Thayer: Pathology and Pathologic Anatomy.........941
Schmidt: Genitourinary and Venereal Diseases .............379
de Schweinitz: Diseases of the Eye .......................535
Scudder: Treatment of Fractures .......................463
Skinner: Therapeutics of Dry Hot Air ...................698
Sobotta-Huber: Histology and Microscopic Anatomy........940
Stelwagon: Diseases of the Skin.298
Sultan: Atlas of Abdominal Hernias....................377
Szymonowicz-MacCallum: Textbook of Histology...........943
Tanner: Memorandum on Poisons .......................703
Taylor: Physician's Account Book .......................384
Thayer: Compend of Special Pathology ..................466
Thompson: Practical Dietetics..146
Thompson: Practical Medicine .864

Transactions:

American Association of Obstetricians and Gynecologists, Vol. XIV., 1901.149
American Gynecological Society, 1902..............782
American Laryngological Association, 1902 .......781
American Medico-Psychological Association, 1902 .783
American Surgical Association, 1902 .............780
Medical Society of the State of New York, 1902......376
State Medical Association of Alabama, 1902........704
State Medical Association of Texas, 1902. ........704
State Medical Society of Wisconsin, 1902 ........704
Southern Surgical and Gynecological Association, Vol. XIV., 1901 ....227

Treat: International Medical Annual, 1903..................944
Tuttle; Diseases of the Anus, Rectum, etc ................380

PAGE.

Tyson: Examination of Urine..541
Ultzmann: Genitourinary System in Male ................224
Van Schaick: Regional Minor Surgery ....................702
Vaughan-Novy: Cellular Toxins.301
von Noorden: Metabolism and Nutrition. I. and II.... 780, 864
von Noorden: Metabolism and Nutrition. III..............945
Warren-Gould: International Textbook of Surgery ........462
Wharton: Minor Surgery and Bandaging ..................149
Wharton: Practice of Surgery..621
Williams: Textbook of Obstetrics.........................775
Witthaus: Manual of Chemistry.376
Wood: Medical Record Visiting List for 1903 ...............383
Woolsley: Applied Surgical Anatomy .................304

Rheumatism, A Real Cure.........132
Acute ................425
Muscular.............131
Roosevelt, Injury to President......232
Ruffner, E. L., Multiple Abscess of Liver ......................... 901
Russell, William L, Diagnosis of General Paralysis of the Insane...723
Ryerson, G. Sterling, My Experiences in War—a Contrast, 1885–1900 ... 1

SAINT LOUIS and the Exposition of 1904................895
Saliva, Quantity of Thiocyanate in Human .................120
Saliva, Alkaline..................206
Samples, Distribution of ...........691
Sanitary Work in Cuba............691
Saxe, G. A. de Santos, Clinical Memoranda on Cough.................881
Scabies Ointment, Liebrich's.........914
Sherry's Bill Signed ...............853
Snow, Irving M., Injuries and Infections of Newly-born Children .....................904
Sargent F., Points in the Treatment of Catarrhal Deafness................389

**Society Meetings:**

American Academy of Medicine.775
American Association of Medical Editors .................695
American Association of Obstetricians and Gynecologists .... ...................61, 217, 936

PAGE.

American Dermatological Association ....................144
American Electro-therapeutic Association .........64, 144, 935
American Medical Association, .........................453, 774
American Medico-Psychologic Association.................617
American Public Health Association .......................369
American Röntgen Ray Society, ....................144, 370, 455
American Urological Association .........................695
Buffalo Academy of Medicine, ................65, 219, 294, 372, 456, 529, 617, 696, 774, 858
Buffalo General Hospital Alumni Association.................371
Congress of American Physicians and Surgeons (Sixth)....371, 858
District Branch New York Medical Association, Fourth ......935
Erie County Medical Association...........143, 452, 695, 935
Fourteenth International Medical Congress ........64, 452, 616
Homeopathic State Medical Society.........................144
Medical Association of Central New York .............297, 370
Medical Society of Missouri Valley ....................144, 618
Medical Society of the County of Chautauqua...........63, 934
Medical Society of the County of Erie ............455, 529, 857
Medical Society, State of New York ...................218, 456
Mississippi Valley Medical Association.................144, 370
National Association of U. S. Pension Examining Surgeons ....................64, 695, 773
National Confederation of State Medical Examining and Licensing Boards .........696, 857
New York State Medical Association..............63, 297, 371
Oregon State Medical Society ..297
Pan-American Medical Congress, Fourth....................935
Roswell Park Medical Club.....218
Sanitary Congress.............774
Southern Surgical and Gynecological Association..143, 296, 368
Tri-State Medical Society, Alabama, Georgia and Tennessee .......................218
Vermont Society Study of Tuberculosis ..................297
Western Ophthalmologic Association .....................617
Western Surgical and Gynecological Association ..........370
Wyoming County Medical Association ....................529

**Society Proceedings :**

Alumni Association, Medical Department University of Buffalo.908
American Association of Obstetricians and Gynecologists ....270
Buffalo Academy of Medicine, ..110, 201, 350, 592, 761, 907, 936
Section on Medicine..350, 351, 515, 591, 679, 838, 904
Section on Pathology......517
Medical Association of Central New York..................347
Medical Society of the County of Erie.....48, 117, 587, 677, 762

Specialty, the Surgical ..............313
State Consumption Hospital........850
Stephenson, F. H., Detention Hospital for Acute Mental Diseases... 33
Stimulation.......................799
Streptococci, Further Investigations Concerning...................914
Suppuration, Causes of ...........583
Surgeons of N. Y. C. & H. R. R. R..691

TELEPHONE, Does it Transmit Disease? .................408
Testicle, Tuberculous Infection of...................127
Testimony, Medical Expert.........566
Therapeutic Truths................597
Thymus, Proteids of...............122
Toast, How to Make ..............914
Tonic Medication ................594
Toxin, Artificial Modifications of....122
Truth.........................851
Tuberculosis, Early Diagnosis of Pulmonary ............ 77
In Childhood.........427
Inhalations of Antiseptics in..............429
Surgical..............256

URIC ACID, Its Sources and Effects ....................123
Urine, Relief of Retention of .......430
Study of Without Microscope.195
Uteri, Carcinoma of the Cervix .....411
Uterine Fibroids..................504
Uterus and Vagina, Labor in Double.513
Uterus, Curetment of..............423

PAGE.
VIRCHOW, RUDOLF, an Appreciation ... 656
Von Ruck, Silvio, Early Diagnosis of Pulmonary Tuberculosis ... 77

WAR, My Experiences in ... 1
Water Supply, Contaminated ... 365
Water Supply and Sewerage of Buffalo ... 809
Westinghouse, G. H., Acetezone in Typhoid Fever ... 40
Whipple, Electa B., Work of Physicians' League ... 29
Whooping Cough, Treatment of ... 913

PAGE.
Wilhelmina, Physicians to Queen ... 57
Williams, Henry T., Labor With Double Uterus and Vagina ... 513
Wilson, Nelson W., Saint Louis and the Exposition of 1904 ... 895
Woehnert, A. E., Blood in Relation to Surgical Diagnosis ... 174
Wounds, Gunshot in Civil Life ... 11

YGDRASIL, In the Shade of ... 769

ZIMMER, JOHN, Unusual Causes of Lead Poisoning ... 393

